# A pilot study on the stability of toluene in blood from workers

**DOI:** 10.1186/1745-6673-7-24

**Published:** 2012-12-02

**Authors:** Masanori Ogawa, Teppei Sasahara

**Affiliations:** 1 Health Service Center, Jichi Medical University, 3311-1, Yakushiji, Shimotsuke-shi, Tochigi, 329-0498, Japan; 2 Division of Bacteriology, Jichi Medical University, 3311-1, Yakushiji, Shimotsuke-shi, Tochigi, 329-0498, Japan

**Keywords:** Stability, Toluene, GC/MS, Medical examination, Volatile organic solvent

## Abstract

**Background:**

Biological monitoring is used to assess toluene exposure in medical examinations. The American Conference of Industrial Hygienists, Japanese Society for Occupational Health and Deutsche Forschungsgemeinschaft have proposed various biological exposure determinants, such as toluene in blood and urine, and o-cresol in urine. Toluene in blood is a common biomarker among them. Toluene is a volatile organic solvent; therefore, sample preservation under appropriate conditions before measurement is necessary. However, little study has been done on the stability of toluene in workers’ blood samples under conditions simulating those of a medical examination.

**Finding:**

We carried out a pilot study on the stability of toluene in blood from humans, according to different methods of sample preservation. Toluene in blood was analyzed by head space-gas chromatography/mass spectrometry. The sealing performance of the vial was examined by using toluene-added blood and the stability of toluene in blood according to the preservation period was examined by using blood from toluene-handling workers, which was collected with vacuum blood tubes. The sealing performance of the headspace vial used in this study was good for three days and toluene in blood in tubes from workers was stable at least within 8 hours up to blood packing at 4°C.

**Conclusion:**

We could propose that the collected blood need only be transferred into headspace vials on the collection day and analyzed within a few days, if the samples are preserved at 4°C. Our data size is limited; however, it may be considered basic information for biological monitoring in medical examinations.

## Introduction

To assess the toluene exposure of workers, various biomarkers are recommended. In the past, hippuric acid (HA) was one of the biomarkers of toluene exposure. However, the dietary intake of benzoic acid, which is present in certain acidic foods and used as food preservatives, influences the urinary HA concentration [1]. Moreover, the consumption of coffee and green tea can result in an overestimation of urinary HA concentrations in the workers exposed to toluene [2,3]. It has also been reported that urinary HA cannot be used to assess the exposure to low concentrations of toluene owing to the effect of its background concentration [1]. The occupational exposure limits set by the Japanese Society for Occupational Health (JSOH), the biological exposure indices set by the American Conference of Industrial Hygienists and the biological tolerance values set by the Deutsche Forschungsgemeinschaft (DFG) the American Conference of Industrial Hygienists (ACGIH) do not use urinary HA to monitor toluene exposure [4-6]. Various biomarkers, such as toluene in blood and urine, and o-cresol in urine, are indicated as markers of toluene exposure from these organizations; toluene in blood is a common indicator [5-7]. Toluene is a volatile organic solvent; therefore, it is thought that sample preservation under appropriate conditions before measurement is necessary.

There are reports about the stability of toluene in urine or toluene-added blood [8-10]; however, a study on the stability of toluene in blood collected from humans, under conditions simulating those of a medical examination, is lacking. In this study, we carried out a pilot study on the stability of toluene in blood collected from toluene-handling workers according to sample preservation conditions.

## Methods

The participants in this study were 11 healthy toluene-handling male workers in two factories (age range, 23–59 years). The concentration of toluene in their workplace was at all times below 20 ppm, which is the TLV adopted by ACGIH [7]. They were working during the daytime from Monday to Friday.

Prior to the analysis of toluene in blood from workers, the sealing performance of the headspace vial used in this study according to time lag from sampling to measurement was studied as follows. Five toluene-added blood samples were prepared and 1 ml of blood from each sample was transferred to a headspace vial. The corresponding silicone septa and aluminum top were put in place, and a crimper was used to seal it. Blood was analyzed on the collection day, the next day and the second day after the sampling day (Days 1, 2 and 3). The cap was tightly sealed on each vial.

In order to study the stability of toluene in blood, total 6 ml of blood was collected from each worker at the end of a Thursday shift, using four EDTA-2Na-containing vacuum blood tubes (Terumo, Japan). The procedures were as follows. From one tube, 1 ml of blood was immediately transferred to a headspace vial and the cap was tightly sealed on each vial. The other tubes were kept at 4°C for 3, 5 and 8 hours, and 1 ml of blood was transferred to a headspace vial after those times. The vial was capped tightly and kept at 4°C before measurement. All vials were kept at 4°C after packing and analyzed on the next day. The sample handling from collection to packing was carried out by the same person. Moreover, these handlingw were done in the same place.

Toluene in blood was analyzed by head space-gas chromatography/mass spectrometry (HS-GC/MS) (Agilent, USA) with 10 ml glass vials (Agilent, USA). The operating conditions of GC/MS are shown in Table 1. The lot number of toluene used as standard solution was the same in all analysis and the samples were analyzed by the same person.

**Table 1:** Operating conditions of GC/MS

	
Column	DB-WAX 60 m × 0.32 mm × 0.25 μm
Oven	50°C (3 min) → 10°C/min → 160°C° → 20°C/min → 200°C (2 min)
Injection dose	10 μL
Split ratio	10:1
Detector	GC-MS
Ion source temperature	230°C
Quadrupole temperature	150°C

This study was approved by the Ethics Committee of Jichi Medical University. All subjects gave their informed consent.

Statistical analyses were performed using StatView 5.0 (SAS Institute Inc., Cary, NC, USA). The sealing performance of this headspace vial according to time lag from sampling to measurement and the differences of the stability of toluene in blood according to the preservation period were compared by Friedman test. Results were considered statistically significant if p values were <0.05.

## Results

**Figure 1:**
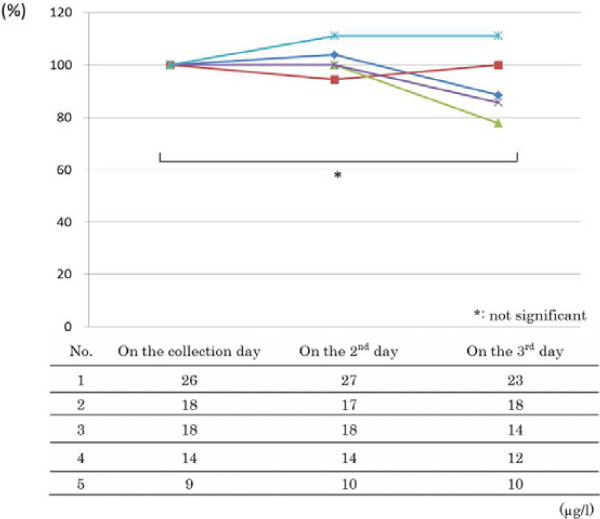
**The toluene level in toluene-added blood according to the time lag between packing and measurement.** The toluene level in toluene-added blood according to the time lag between packing and measurement, setting the toluene levels on the collection day to be 100%.

The toluene levels in toluene-added blood according to the time lag between packing and measurement are shown in Figure 1. We set the toluene levels on the collection day to be 100% and compared the variation according to the measurement days. There was no significant difference in blood toluene levels among on the collection day (Day 1), the next day (Day 2) and the 2nd day after sampling (Day 3), as determined by Friedman test.

**Figure 2:**
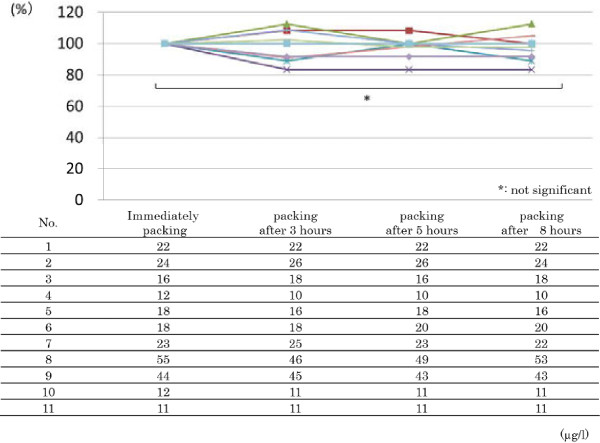
**The change of toluene level in blood from workers according to the preservation period.** The toluene level in blood from workers according to the preservation period, setting the toluene levels immediately after packing to be 100%.

The toluene level in the blood of workers ranged from 11 ppm to 55 ppm in the immediately packed vials. These levels are all under occupational exposure limits set by JSOH, ACGIH and DFG. We set these toluene levels to be 100% and compared the variation according to the preservation period. Changes in toluene levels are shown in Figure 2. There are no significant differences in the blood toluene levels according to the preservation period, as determined by Friedman test.

## Discussion

Toluene is a volatile organic compound [11], therefore, it is important to know the stability of blood toluene under the various preservations methods used previously for medical examination.

There are some reports concerning the stability of toluene in toluene-added blood in blood-collecting vessels. However, studies using blood from toluene-exposed workers in the context of medical examinations are lacking. Therefore, in this study we measured toluene in blood from toluene-exposed workers using four protocols, simulating an actual medical examination. In such circumstances, it is suspected that blood samples are not handled immediately after sampling on the spot in most cases. Therefore, we prepared three protocols, in which blood was transferred to vial after preservation for 3, 5 and 8 hours at 4°C, in addition to packing immediately after sampling.

It was demonstrated that the vials fitted with either Teflon- or aluminum- lined septa offer the best sample stability at both room temperature and 4°C for a long time if the vials are filled with blood. Moreover, it has also been reported that bottles equilibrated at 4°C with a large or a small headspace during storage showed no significant difference in toluene concentrations [9]. Therefore, we transferred only 1 ml of blood to headspace vials in this study because the collection of more blood than necessary is invasive, and we kept the headspace vials at 4°C until measurement. Our data indicate that the sealing performance of this kind of vial is high and the toluene concentration does not change for at least for 3 days if we seal the vial tightly. It is suspected that there are situations in which the samples are not analyzed on the collection day in actual medical examinations, depending on the number of samples. Therefore, in this study we analyzed the samples from toluene-handling workers on the next day, based on our result and the suspected situation.

Saker et al. [10] preserved toluene-added blood in blood-collecting vessels covered with Parafilm® for 1, 2 and 7 weeks at 24°C, 4°C and −18°C and measured blood toluene concentrations using a headspace vial by HS-GC/MS, which is the same method we used. They found that the blood toluene levels did not change significantly at 24°C, 4°C and −18°C for one week. On the contrary, in another study using toluene-added blood, Gill et al. [9] described that the sample at 4°C showed little overall change while the sample at room temperature showed a drop in concentration of about 13% within 1 hour in an open container exposed to the atmosphere. Therefore, we kept blood-collecting vessels at 4°C after blood sampling until we packed the blood into head space vials, and our data show that blood toluene concentrations did not change significantly within 8 hours up to blood packing. Although we did not examine the stability over 8 hours after collection to packing, we can propose that technicians only have to transfer the blood to vials on the blood collection day if the samples are preserved at 4°C.

In the HS-GC/MS analysis, there is also a concern about sample measurement error. Miyaura et al. [12] reported the precision of blood toluene analysis by HS-GC/MS using almost the same devices as those used in this study and mentioned that the measurement error was within 5%. In this study, the sample handling from collection to packing and the analysis were done under the same condition in each sample. Moreover, the standard solution of toluene in HS-GC/MS had the same lot number. Therefore, we assume that the measurment error will not affect these findings.

We can say that toluene in blood is stable from this and previous studies. We suspect the reason why toluene is stable in blood is due to the influence of the hydrophobic matrix of the biological fluid. Toluene is a hydrophobic substance; therefore, it is suspected that toluene bonds with the lipids in blood, and that this hydrophobic effect explains why toluene in blood is not more evaporative during storage [13,14].

JSOH and ACGIH have adopted both toluene in blood and urine as biomarkers [5-7]. Blood sampling is invasive to workers and urine sampling is not at all. Therefore, urine seems more suitable than blood for an indicator of toluene exposure in a medical examination [15,16]. It is indicated by JSOH that the sampling time of blood or urine for the assessment of toluene exposure is the same-‘within 2 h prior to end of shift at end of work week’. According to the ACGIH document [7], the sampling time for urine is the ‘end of shift’; on the contrary, the sampling time for blood is ‘prior to last shift of the weekend’. Although urine sampling is more convenient and less invasive than blood sampling to screen for toluene exposure, there may be situations in which blood sampling for toluene is more useful, for example when the medical examination must be carried out prior to a shift. Our data is limited; however, it provides basic information for medical examinations because we use blood from actual toluene-handling workers in the simulated conditions of a medical examination.

## Competing interests

The authors declare that they have no competing interests.

## Authors’ contributions

MO participated in the design of the study and the collection of samples, performed the statistical analysis and draft the manuscript. TS participated in the collection of samples. Both authors read and approved the final manuscript.
